# A Complete Mitochondrial Genome Sequence from a Mesolithic Wild Aurochs (*Bos primigenius*)

**DOI:** 10.1371/journal.pone.0009255

**Published:** 2010-02-17

**Authors:** Ceiridwen J. Edwards, David A. Magee, Stephen D. E. Park, Paul A. McGettigan, Amanda J. Lohan, Alison Murphy, Emma K. Finlay, Beth Shapiro, Andrew T. Chamberlain, Martin B. Richards, Daniel G. Bradley, Brendan J. Loftus, David E. MacHugh

**Affiliations:** 1 Smurfit Institute of Genetics, Trinity College, Dublin, Ireland; 2 Animal Genomics Laboratory, School of Agriculture, Food Science and Veterinary Medicine, College of Life Sciences, University College Dublin, Dublin, Ireland; 3 Conway Institute of Biomolecular and Biomedical Research, University College Dublin, Dublin, Ireland; 4 Henry Wellcome Ancient Biomolecules Centre, Department of Zoology, Oxford University, Oxford, United Kingdom; 5 Department of Archaeology, University of Sheffield, Sheffield, United Kingdom; 6 Institute of Integrative and Comparative Biology, Faculty of Biological Sciences, University of Leeds, Leeds, United Kingdom; University of Otago, New Zealand

## Abstract

**Background:**

The derivation of domestic cattle from the extinct wild aurochs (*Bos primigenius*) has been well-documented by archaeological and genetic studies. Genetic studies point towards the Neolithic Near East as the centre of origin for *Bos taurus*, with some lines of evidence suggesting possible, albeit rare, genetic contributions from locally domesticated wild aurochsen across Eurasia. Inferences from these investigations have been based largely on the analysis of partial mitochondrial DNA sequences generated from modern animals, with limited sequence data from ancient aurochsen samples. Recent developments in DNA sequencing technologies, however, are affording new opportunities for the examination of genetic material retrieved from extinct species, providing new insight into their evolutionary history. Here we present DNA sequence analysis of the first complete mitochondrial genome (16,338 base pairs) from an archaeologically-verified and exceptionally-well preserved aurochs bone sample.

**Methodology:**

DNA extracts were generated from an aurochs humerus bone sample recovered from a cave site located in Derbyshire, England and radiocarbon-dated to 6,738±68 calibrated years before present. These extracts were prepared for both Sanger and next generation DNA sequencing technologies (Illumina Genome Analyzer). In total, 289.9 megabases (22.48%) of the post-filtered DNA sequences generated using the Illumina Genome Analyzer from this sample mapped with confidence to the bovine genome. A consensus *B. primigenius* mitochondrial genome sequence was constructed and was analysed alongside all available complete bovine mitochondrial genome sequences.

**Conclusions:**

For all nucleotide positions where both Sanger and Illumina Genome Analyzer sequencing methods gave high-confidence calls, no discrepancies were observed. Sequence analysis reveals evidence of heteroplasmy in this sample and places this mitochondrial genome sequence securely within a previously identified aurochsen haplogroup (haplogroup P), thus providing novel insights into pre-domestic patterns of variation. The high proportion of authentic, endogenous aurochs DNA preserved in this sample bodes well for future efforts to determine the complete genome sequence of a wild ancestor of domestic cattle.

## Introduction

The now-extinct aurochs (*Bos primigenius*), which ranged throughout much of Eurasia and Northern Africa during the late Pleistocene and early Holocene, is widely accepted as the wild ancestor of modern cattle [1], [2]. Archaeological evidence shows that domestication of this formidable animal occurred independently in the Near East and the Indian subcontinent between 10,000–8,000 years ago, giving rise to the two major domestic taxa observed today — humpless *Bos taurus* (taurine) and humped *Bos indicus* (zebu), respectively [3], [4]. This is confirmed by genetic analyses of matrilineal mitochondrial DNA (mtDNA) sequences, which reveal a marked differentiation between modern *B. taurus* and *B. indicus* haplotypes, demonstrating their derivation from two geographically- and genetically-divergent wild populations [5].

More recently, the fine-scale phylogeographic structure of extant bovine mtDNA sequence variation has been elucidated. The majority of taurine mtDNA sequences cluster within macro-haplogroup T, which consists of six sub-haplogroups (T, T1, T2, T3, T4 and T5) [6], [7], [8]. Geographic distribution of these sub-haplogroups has provided evidence for the derived Near Eastern origin of European *B. taurus* and lent support for separate domestications of African and North East Asian *B. taurus* cattle [7], [8]. *B. indicus* mtDNA sequences are highly divergent from *B. taurus* sequences, and fall within macro-haplogroup I. This macro-haplogroup is further subdivided into the I1 and I2 sub-haplogroups, which display some geographic partitioning across South Asia [9], [10], [11].

DNA sequences retrieved from ancient cattle have also shed light on the pre-domestication history of the *Bos* spp. lineage. Partial mtDNA control region and cytochrome b gene (*CYTB*) sequences from Central and Northern European aurochsen samples belong predominantly to a distinct haplogroup (designated ‘P’), which diverged from macro-haplogroup T prior to domestication, though substantially later than the branching leading to macro-haplogroup I [8], [12], [13], [14]. A single exception, a sample from the Early Neolithic German site of Eilsleben, falls outside the clade containing haplogroup P and macro-haplogroup T, and has been assigned the novel haplotype E [13]. A contrasting picture is seen in Italy, where all ancient aurochsen mtDNA control region sequences to date have been assigned to macro-haplogroup T [15]. Molecular clock estimates based on ancient sequences indicate that haplogroup P underwent an expansion between 10,050 and 30,230 years prior to sample deposition, or approximately 16,000 to 36,000 years before present (yBP). This is consistent with a Late Glacial repopulation of Northern and Central Europe from Iberian and/or Balkan refugia [13]. Expansion within haplogroup P seems to pre-date expansion within the sub-haplogroups of macro-haplogroup T, which appears to have occurred around the time of domestication in the Near East [6], [8], [16].

The observation that modern European *B. taurus* belong almost exclusively to macro-haplogroup T together with the absence of haplogroup P sequences in these animals suggests that there was no major maternal contribution to the European domestic cattle genetic pool from locally recruited wild cattle [8], [13], [17]. However, the recent reporting of two novel, albeit rare, putative aurochs sequences (haplogroups Q and R) in modern animals sampled in Italy, together with the detection of a haplogroup P sequence in a modern taurine animal support at least some local adoption of wild aurochsen matrilines [6], [16]. Moreover, the detection of pre-Neolithic macro-haplogroup T sequences in European aurochs samples has led some authors to argue that this haplogroup was not restricted to the Near East, and that wild haplogroup T females may have been incorporated locally into the European domestic pool [15]. In contrast to mtDNA studies, analyses of paternally-inherited Y-chromosome haplotypes are equivocal on whether male aurochsen contributed to European *B. taurus* [18], [19].

The recent advent of high-throughput DNA sequencing technologies, also referred to as ‘next-generation sequencing’ (NGS) technologies, is heralding a new era for ancient DNA studies [20]. Such investigations have already generated considerable DNA sequence information from extinct mammals, including complete mtDNA genomes from ancient woolly mammoth, thylacine, rhinoceroses and hominid samples, giving new insight into their evolutionary history [21], [22], [23], [24], [25], [26], [27], [28], [29]. Future research is expected to reveal functional genetic polymorphisms, such as that previously reported for the Neanderthal mtDNA genome [26]. NGS technologies also enable the quantification of *post-mortem* chemical modification and contamination with modern genetic material, the two major sources of error in ancient DNA sequencing [21], [26], [30], [31], [32], [33], [34].

In 2001, we reported DNA sequence variation in the most variable 240 base pair (bp) fragment of the mtDNA control region from four British aurochs samples [8]. In this and subsequent laboratory studies performed by us, a single aurochs humerus bone sample (laboratory code CPC98) consistently yielded high-quality *B. primigenius* mtDNA sequences allowing phylogenetic placement of this sample within haplogroup P [13], [14]. This sample was excavated in 1998 from Carsington Pasture Cave in Derbyshire, England, and has been radiocarbon dated to 6,738±68 calibrated (cal.) yBP. This pre-dates the start of the Neolithic period in Britain (5,900–5,580 cal. yBP) [35] and the earliest British macro-haplogroup T-carrying bone sample [13], giving a secure basis for its classification as *B. primigenius*.

Here, we present the complete mtDNA genome sequence of the CPC98 sample using a combination of conventional polymerase chain reaction (PCR)/Sanger DNA sequencing and a direct high-throughput sequencing approach (Illumina Genome Analyzer). To our knowledge, this study represents the first description of the complete mtDNA genome sequence of an archaeologically-verified aurochs. We have analysed the complete aurochs mtDNA genome sequence together with previously published complete mtDNA sequences generated from modern bovine samples. We discuss our findings in light of the current understanding of the evolutionary history of domestic cattle. In addition, we note that the high proportion of authentic aurochs DNA sequences preserved in this sample offers promise for future efforts to obtain a complete *B. primigenius* genome sequence.

## Results

### Summary of Sanger mtDNA Sequencing

A total of 31 primer pairs were designed, based on the GenBank *B. taurus* mtDNA reference sequence (GenBank accession no. V00654) [36] to generate partially-overlapping polymerase chain reaction (PCR) amplicons, with an average length of 646 bp, spanning the complete sequence of the aurochs mtDNA genome. Successful PCR amplifications were produced from multiple independent CPC98 DNA extractions (ranging from two to seven independent DNA extractions) for all 31 amplicons using a multiplex PCR approach [37], and were sequenced bi-directionally using the conventional Sanger-based methodology. All independent PCR amplicons were sequenced between four and 17 times. Alignment of the DNA sequences (with PHRED scores ≥16) with the *B. taurus* reference mtDNA sequence allowed overlapping sequences to be identified, and assembled into a contiguous, complete CPC98 mtDNA sequence consisting of 16,338 bp. Sequencing depth across the contiguous 16,338 bp mtDNA genome ranged between 2× and 19× with an average of 7.9×. There is an apparent discrepancy in the sequencing depth for each mtDNA nucleotide (ranging from 2× to 19×) and the number of times independent amplicons were sequenced (ranging from 4× to 17×). This is due to the overlapping nature of several of the PCR amplicons and also the filtering of nucleotides displaying low PHRED scores (≤16). Detailed information for each of the PCR amplicons used for Sanger-based sequencing is provided in the supporting information (Table S1).

### Summary of Illumina Genome Analyzer Sequencing and mtDNA Sequence Assembly

A total of 49,125,583 Illumina Genome Analyzer (GA) single sequence reads, each 36 nucleotides in length, were produced from three independently prepared CPC98 Illumina GA libraries and sequenced across 14 lanes of three flow cells. 13,292,821 reads (27.06%) were identified as consisting largely or entirely of Illumina GA sequencing adaptor sequence and were excluded.

Of the remaining non-Illumina GA adaptor reads, sequence alignment analysis showed that 8,053,754 reads (22.48% of the non-adaptor reads), comprising 289.93 Mb, mapped with high-confidence (PHRED score ≥30) to Btau4.0 build of the *B. taurus* reference nuclear genome sequence (http://genome.ucsc.edu/). Screening for potential duplicate read sequences (reads from the same Illumina GA library, mapping to the same base position on the same strand) found only 100,922 such reads (1.25% of the total non-adaptor reads aligning to the bovine genome). This yields a conservative total of 7,952,832 non-duplicate, bovine genome-aligned reads.

5,144 reads (0.06% of bovine genome-aligned reads with potential duplicates included), yielding 185.1 kb, mapped with high-confidence across their whole length to the *B. taurus* reference haplogroup P reference mtDNA sequence (GenBank accession no. DQ124389). Removal of all possible duplicate reads left 4,108 non-duplicate reads, giving 147.9 kb of sequence. These figures give a ratio of nuclear DNA reads to bovine mtDNA reads of 1,930∶1. When genome size is considered, and assuming a bovine diploid nuclear genome size of 5.74 gigabases [38], we obtained an estimate of the ratio of mtDNA genome sequences to nuclear genome sequences of 182∶1 in the CPC98 bone sample.

The 4,108 non-duplicate mtDNA reads were assembled into an alignment and the consensus was determined for each nucleotide position. The total length of the Illumina GA-generated mtDNA consensus sequence where ≥ two-fold coverage was obtained for each nucleotide position was 15,339 bp. The average read depth across these 15,339 bp was 9.6×. Interspersed within this sequence were 51 regions not covered by any reads or covered at a read depth of 1× only. For these sequence-poor regions, no sequence information was obtained for 420 nucleotide positions of the reference sequence and 581 nucleotide positions were covered at a read depth of 1×. The identity of the missing nucleotides and those nucleotides with 1× coverage was determined via comparison with the Sanger-generated CPC98 mtDNA sequence.

Comparison of the Illumina GA-generated 15,339 bp mtDNA consensus sequence with the Sanger sequence revealed a total of three nucleotide mismatches at positions 2,614, 10,045 and 16,121 (note: all nucleotide positions reported in this study are numbered according to the *B. taurus* mtDNA reference sequence, GenBank accession no. V00654). Two of these (nucleotide positions 2,614 and 10,045) were covered at a read depth of only 2× and exhibited ambiguous nucleotides. The corresponding positions in the Sanger sequence were unambiguous and were used for assembly of the final consensus mtDNA sequence. The final mismatch at position 16,121 displayed heteroplasmy and is discussed later. A summary of the DNA sequences generated using the Illumina GA for the CPC98 sample, including nuclear and mtDNA genomic data, is detailed in Table 1.

**Table 1:** Summary of Illumina GA sequencing data for the CPC98 aurochs sample.

Summary of Illumina GA sequencing data for CPC98 aurochs femur bone	
**Overall Illumina GA sequencing summary**	
Total number of sequence reads generated from CPC98	49,125,583
Total number of partial/complete Illumina GA adaptor sequences detected and excluded from analysis	13,292,821
Total number of non-adaptor Illumina GA reads generated from CPC98	35,832,762
Total number of base pairs (bp) sequenced from CPC98 (excluding Illumina GA adaptor sequences)	1,289,979,432 bp
Total number of non-adaptor sequence reads mapping to the bovine genome (% of total non-adaptor CPC98 reads)	8,053,754 (22.48%)
Total number of base pairs mapping to bovine genome	289,935,144 bp
Total number of non-adaptor reads mapping to the bovine genome and not to human genome (% of total non-adaptor CPC98 reads)	7,868,524 (21.96%)
Total number of base pairs mapping to bovine genome and not to human genome	283,266,864 bp
Total number of sequence reads mapping to the bovine and human genomes (% of total non-adaptor CPC98 reads)	185,097 (0.52%)
Total number of base pairs mapping to bovine and human genomes	6,663,492 bp
Total number of sequence reads mapping to the human genome and not the bovine genome (% of total non-adaptor CPC98 reads)	48,555 (0.14%)
Total number of base pairs mapping to human genome and not the bovine genome	1,747,980 bp
**CPC98 mtDNA genome information**	
mtDNA haplogroup of CPC98	P
Total number of reads mapping to bovine haplogroup P mtDNA reference sequence DQ124389 (% of total non-adaptor CPC98 reads)	5,144 (0.06%)
Total number of potential duplicate reads mapping to bovine haplogroup P mtDNA sequence	1,036
Total number of non-duplicate reads mapping to bovine haplogroup P mtDNA sequence (% of total non-adaptor CPC98 reads)	4,108 (0.05%)
Total number of non-duplicate base pairs mapping to bovine haplogroup P mtDNA reference sequence	147,888 bp
Size of Illumina GA-generated CPC98 mtDNA genome (where ≥2× sequencing coverage obtained)	15,339 bp
Mean sequencing depth of Illumina GA-generated CPC98 mtDNA genome	9.6×
Size of Illumina GA and Sanger consensus mtDNA genome	16,338 bp
Mean sequencing depth of combined Illumina GA-generated CPC98 mtDNA genome	16.9×
Number of nucleotide differences between CPC98 and V00654 mtDNA sequences (ti/tv/indels)	71 (62/7/2)
Number of nucleotide differences between CPC98 and DQ124389 mtDNA sequences (ti/tv/indels/undetermined*)	22 (19/0/2/1)

*This includes a putative substitution at nucleotide position 15,714 which was called as an ‘N’ in sample DQ124389. ti (transitions); tv (transversions); indels (insertion/deletions).

Overall, combined analysis of the Illumina GA and Sanger sequences yielded a final mtDNA consensus assembly of 16,338 bp. The lowest sequence coverage was 2×, for just one nucleotide position (nucleotide position 67). Eighty-nine positions were covered at 3× and all other positions were covered at 4× or greater. The combined average sequence depth across all 16,338 bp was estimated at 16.9×. This mtDNA consensus sequence was used for all subsequent analyses described here.

### Estimates of CPC98 Contamination with Modern DNA

#### (a) Contamination with modern bovine DNA sequences

To estimate the level of sequence contamination of the CPC98 Illumina GA libraries with modern bovine DNA, we first catalogued mtDNA single nucleotide polymorphisms (SNPs) distinguishing haplogroup P sequences from modern macro-haplogroup T, Q, R and I sequences. We then scanned each of the 4,108 non-duplicate reads mapped to the bovine mtDNA for the presence or absence of these haplogroup P-specific alleles. For this, 130 modern macro-haplogroup T, six haplogroup Q, four haplogroup R and seven haplogroup I complete mtDNA sequences retrieved from GenBank (http://www.ncbi.nlm.nih.gov) were aligned along with the published modern haplogroup P sequence (GenBank accession DQ124389) and the CPC98 mtDNA consensus sequence obtained from Sanger and Illumina GA sequencing. In total, 18 SNPs discriminating the two haplogroup P sequences from the haplogroup T, Q and I sequences were identified. When haplogroup R sequences are included in this analysis, this figure reduces to 16 SNPs. Nucleotide calls for these positions were obtained from the individual Illumina GA sequence reads.

A total of 117 CPC98 reads spanned the 18 SNPs differentiating the haplogroup P sequences from haplogroup T, Q and I sequences. When haplogroup R sequences are considered, 110 reads span the 16 haplogroup P-defining substitutions (the CPC98 consensus allele at position 6,160 and 16,264 are the same as the haplogroup R). In both cases, only one of these reads carried the non-haplogroup P allele: a ‘C’ allele at nucleotide position 7,952, for which the haplogroup P consensus allele is a ‘T’. Considering this T-to-C mismatch at position 7,952 as a possible modern haplogroup T, R, Q or I contaminant in the Illumina GA CPC98 mtDNA sequence data we obtain an upper limit for modern bovine contamination of 1/117 (0.85%) (Table 2).

**Table 2:** Estimates of contamination with modern bovine DNA sequences.

	SNP allele	A			C			G			T			Illumina GA read depth	CPC98 consensus allele	Nucleotide position in Illumina GA read where mismatch occurs	Possible source of discrepancy
	Macro-haplogroup/Haplogroup	I	Q,R,T	P	I	Q,R,T	P	I	Q,R,T	P	I	Q,R,T	P				
	301				7	140							2	4 x T	T	No	
	1,128	7	140							2				10 x G	G	No	
	2,585						2				7	140		5 x C	C	No	
	4,293				7	140							2	3 x T	T	No	
	4,676	7	140							2				9 x G	G	No	
	5,899	7	140							2				8 x G	G	No	
	7,952				7	140							2	17 x T; 1 x C	T	Yes	Sequencing error/contamination
	8,236						2				7	140		4 x C	C	No	
	8,358				7	140							2	8 x T	T	No	
	10,126				7	140							2	9 x T	T	No	
	11,140	7	140							2				2 x G	G	No	
	12,016						2				7	140		1 x C	C	No	
	13,821	7	140							2				8 x G	G	No	
	14,129	7							140				2	11 x T	T	No	
	14,873			2				7	140					6 x A	A	No	
	15,673						2				7	140		5 x C	C	No	

The nucleotide position (as per the bovine mtDNA reference sequence, GenBank accession no. V00654) of each of the haplogroup P-diagnostic mtDNA SNPs is given in the left-hand column. The SNP allele identities for the I, R, Q and T haplogroups are shown. The numbers represent the number of times that an allele is observed in each of the bovine macro-haplogroups/haplogroups. The haplogroup P allele for the consensus sequence is provided along with the allele and read depth for each of the individual Illumina GA reads spanning the haplogroup P-diagnostic SNPs.

#### (b) Contamination with modern human DNA sequences

We next investigated possible human contamination in the Illumina GA CPC98 reads. As stated previously, 8,053,754 reads (of which only 1.25% are potential duplicates) mapped to the bovine genome (PHRED score ≥30). Further analysis of these bovine genome-aligned sequence reads showed that 7,868,524 reads (21.96% of all non-adaptor reads) mapped to the bovine genome but not the human genome; 185,097 reads (0.52%) mapped to both the bovine and human genomes; and 48,555 reads (0.14%) mapped to the human genome only. 27,779,008 reads (77.52%) contained sequences that could not be aligned to either the bovine or human genomes. These figures provide an upper limit of contamination of CPC98 reads with modern human DNA only of 0.14% (Table 1).

### Estimates of DNA Sequencing Errors for the CPC98 Aurochs Sample

**Figure 1:**
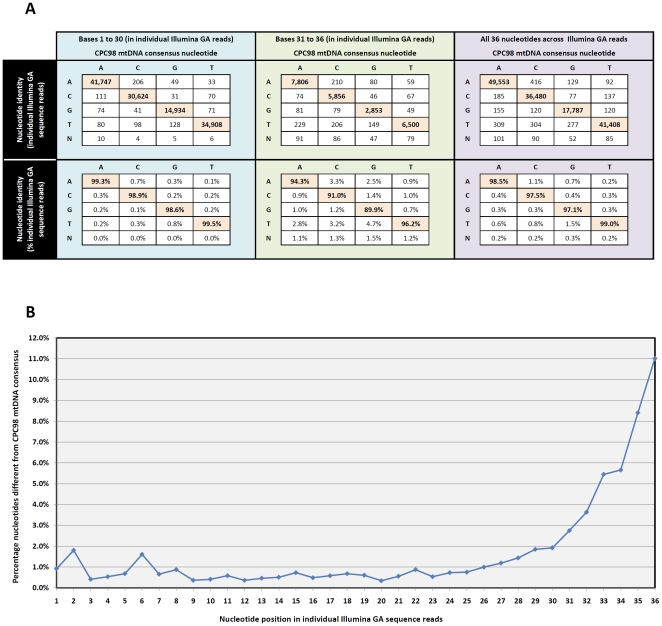
The identity and distribution of DNA nucleotide mismatches in individual Illumina GA reads compared to the CPC98 consensus mtDNA genome. (A) The number and proportion of each nucleotide called in the Illumina GA reads (vertical column) compared to the consensus mtDNA sequence (horizontal column) is presented. (B) Mean percentage of discordant nucleotides for each position across all individual Illumina GA sequence reads.

The extent of DNA sequence error within the individual non-duplicate Illumina GA reads was assessed by comparing all non-duplicate Illumina GA nucleotide calls with the CPC98 consensus mtDNA sequence. 2,321 nucleotide mismatches were observed in a total of 147,549 individual Illumina GA nucleotide calls when compared to the CPC98 consensus sequence, yielding an estimated sequencing error rate of 1.57% (note: this figure excludes the observed heteroplasmy at position 16,121). C-to-T (*n* = 304; 0.81% consensus sequence C nucleotide calls called as T in individual Illumina GA reads) and G-to-A (*n* = 129; 0.70% consensus sequence G nucleotide calls called as A in individual Illumina GA reads) transitions accounted for 433 of the 2,321 identified nucleotide substitutions (18.66%) (Figure 1). C-to-A and G-to-T occur more frequently than expected mismatches caused by *post-mortem* cytosine deamination, namely C-to-T and G-to-A mismatches (1.11% C-to-A *versus* 0.81% for C-to-T and 1.51% G-to-T *versus* 0.70% for G-to-A), known to affect ancient DNA. Further analysis demonstrated that the majority of these detected nucleotide substitutions occur with the six 3′-most nucleotides of the individual Illumina GA 36 nucleotide-long reads—1,329/2,321 (57.30%) substitutions occur within these terminal six nucleotides (Figure 1).

### Sequence Analysis of the CPC98 Aurochs mtDNA Sequence

**Figure 2:**
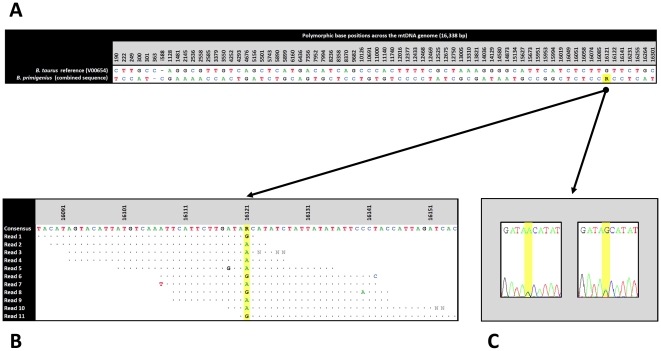
Location of substitutions between the *B. taurus* reference and the *B. primigenius* (CPC98) mtDNA genome sequences and evidence of mtDNA heteroplasmy at nucleotide position 16,121 in the CPC98 aurochs sample. (A) Location of substitutions between the *B. taurus* reference and the *B. primigenius* (CPC98) mtDNA genome sequences. (B) Heteroplasmy detected from analysis of individual Illumina GA reads spanning nucleotide position 16,121. (C) Heteroplasmy at nucleotide position 16,121 detected from analysis of Sanger chromatograms. Nucleotide positions according to the bovine mtDNA reference sequence (GenBank accession no. V00654).

Alignment of the consensus CPC98 mtDNA sequence with the *B. taurus* mtDNA reference sequence (GenBank accession no. V00654) revealed a total of 71 variable nucleotide positions comprising 62 transitions, seven transversions and two indels (Figure 2). This result confirms the strong transitional bias in domestic animal mtDNA sequences as reported in previous studies [8], [11], [13], [14], [16], [17], [39], [40], [41], [42], [43]. Twenty-two of these 71 (30.99%) nucleotide differences occur within the non-coding control region of the mtDNA genome. Of the remaining 49 substitutions, 37 occur in 12 of the 13 mitochondrial protein coding genes, five occur in five of the 22 mitochondrial tRNA genes, and seven occur in the two mitochondrial rRNA genes.

Notably, one nucleotide position in the control region sequence of CPC98 (16,121) is bi-allelic. Seven of the 11 non-duplicate Illumina GA reads spanning this position exhibited an A allele and the remaining four exhibited a G allele, while two potential duplicate reads also exhibited a G allele. The consensus nucleotide was an A/G polymorphism (MAQ program consensus sequence PHRED score = 44). The Sanger sequence data also showed that both A and G alleles were present at this position, as revealed by double chromatophore peaks (8× coverage from four independent DNA extracts). Given the high-confidence sequencing coverage and verification of this bi-allelic SNP from independently-prepared CPC98 DNA extracts using two sequencing technologies, we believe that this finding most probably represents mtDNA sequence heteroplasmy—namely the existence of two or more different mtDNA sequences within the same cell/tissue/individual (Figure 2).

### Phylogenetic Analysis of the CPC98 Aurochs mtDNA Sequence

**Figure 3:**
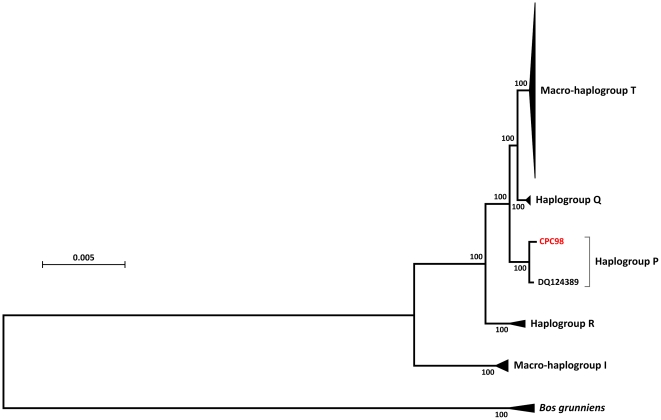
Rooted Neighbor-Joining (N–J) phylogenetic tree detailing the relationships among all available complete bovine haplogroup I, P, Q, R and T mtDNA genome sequences and five yak (*B. grunniens*) mtDNA genome sequences. Evolutionary distances were computed using the Maximum Composite Likelihood method and are in the units of the number of base substitutions per site. Only coding region sequences of the mtDNA genome were used for tree construction (mtDNA nucleotide position 364–15,791). Bootstrap values (1000 replicates) are shown next to the branches. The number of mtDNA sequences within each of the haplogroups is indicated. The haplogroup to which the CPC98 mtDNA genome sequence belongs is highlighted. Five complete mtDNA genome sequences from yak (*B. grunniens*) were used as outgroups.

Phylogenetic analyses indicate that the CPC98 mtDNA sequence belongs to the previously defined aurochs-specific haplogroup P. Achilli and colleagues identified 37 substitutions that have occurred since this haplogroup diverged from the common ancestor with macro-haplogroup T and haplogroup Q [16]. The CPC98 mtDNA sequence possesses 31 of these 37 nucleotide substitutions. Also, the phylogenetic tree presented in Figure 3 constructed from the CPC98 mtDNA consensus sequence, all available complete mtDNA sequences from domestic cattle (*n* = 148) and *B. grunniens* (yak; *n* = 5), shows that the CPC98 mtDNA sequence forms a distinct and well-supported (100% bootstrap support, 1,000 replicates) clade with the only other complete haplogroup P mtDNA sequence DQ124389.

**Figure 4:**
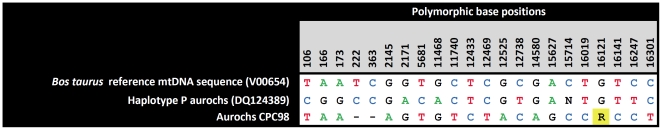
Location of substitutions distinguishing the complete CPC98 consensus mtDNA genome sequence and the other complete haplogroup P sequence (GenBank accession no. DQ124389). Nucleotide positions according to the bovine mtDNA reference sequence (GenBank accession no. V00654).

Haplogroup P is a sister clade of the super-haplogroup QT with a previously estimated divergence time of 71,000 yBP [16]. The DQ124389 sequence was encountered in a modern animal sampled in Korea and was deposited in GenBank in 2006 (Shin and Kim, unpublished data) and subsequently classified as belonging to haplogroup P [14], [16]. Comparison of the consensus CPC98 mtDNA sequence with the DQ124389 mtDNA sequence revealed a total of 22 nucleotide differences, comprising 20 transitions (including two heteroplasmic sites: one at position 15,714 in DQ124389 and one at position 16,121 in CPC98) and two indels (Figure 4). Ten of the 22 nucleotide differences occur in the mtDNA control region, eight in three of the 13 protein-coding genes, two in the 16S rRNA gene, one in the tRNA-Tyr gene, and one in the tRNA-Thr gene.

To estimate the divergence time within haplogroup P, we first counted the number of substitutions [the parameter ‘rho’ (ρ)] between each of the CPC98 and DQ124389 samples and an inferred mutationally-equidistant ancestral sequence and multiplied by the previously used rate estimate of ∼3,172 years per substitution in the coding region of the mtDNA genome (15,428 bp between nucleotide positions 364 and 15,791) [16]. This generated a coalescence time of 19,032 years (95% confidence intervals 9,834-31,215 yBP). This figure however, must be adjusted as the CPC98 and DQ124389 samples are not contemporaneous, but lived 6,738±68 years apart according to radiocarbon dating. Adding half of this time difference gives a coalescence time of 22,401 yBP (95% confidence interval range 13,135-34,652 yBP).

Finally, we calculated genetic diversity statistics for each of the bovine mtDNA haplogroups including the data from the CPC98 sample and additional sequences from previously unanalysed haplogroup T sequences (Table 3). Taking these new data into account, nucleotide diversity (π) is highest in macro-haplogroup I compared to all other haplogroups. The diversity estimate for haplogroup P falls between that generated for macro-haplogroup T and haplogroup R.

**Table 3:** Nucleotide diversity statistics for each of the major *Bos* mtDNA haplogroups.

		Coding region of mtDNA genome (nucleotide positions 364-15,791				Whole mtDNA genome			
Super-haplogroup/Macro-haplogroup/Haplogroup	No. of mtDNAs	No. polymorphic sites	π	σ	tv/ti	No. polymorphic sites	π	σ	tv/ti
Super-haplogroup IRPQT	149	627	0.002129	0.001033	0.07	766	0.002792	0.001347	0.07
Super-haplogroup RPQT	142	474	0.001058	0.000525	0.07	599	0.001567	0.000766	0.08
Super-haplogroup PQT	138	412	0.000793	0.000399	0.08	526	0.001214	0.000598	0.09
Super-haplogroup QT	136	385	0.000724	0.000366	0.07	492	0.001111	0.000549	0.08
Macro-haplogroup I	7	32	0.000926	0.000543	0.14	47	0.001259	0.000727	0.21
Haplogroup P	2	11	0.000713	0.000745	0.00	22	0.001346	0.001377	0.00
Haplogroup R	4	28	0.000907	0.000619	0.00	36	0.001101	0.000744	0.03
Haplogroup Q	6	15	0.000480	0.000302	0.00	27	0.000845	0.000512	0.15
Macro-haplogroup T	130	357	0.000616	0.000315	0.08	460	0.000986	0.000490	0.08

Nucleotide diversity estimates (π) and standard deviations (σ) together with the total number of polymorphic sites for haplogroups I, R, P, Q and T are presented based on coding and complete mtDNA sequences. ti/tv (transition-to-transversion ratios) within each haplogroup are also given.

### DNA Sequence Diversity in Bovine Mitochondrial Protein and RNA Genes

The existence of potential functional nucleotide substitutions unique to haplogroup P was examined by aligning the CPC98 consensus mtDNA sequence with all complete modern cattle mtDNA sequences (*n* = 148) and screening for SNPs that occur in: (1) mitochondrial RNA genes and (2) mitochondrial protein-coding genes causing amino acid replacements. No non-synonymous substitutions were specific to and fixed in haplogroup P. Six of the 31 haplogroup P-defining substitutions present in the CPC98 mtDNA sequence occur in five different RNA genes. Four of these substitutions are unique to haplogroup P (nucleotide position 1,128 in 12S rRNA; 2,585 in 16S rRNA; 12,016 in tRNA-Ser and 15,673 in tRNA-Thr).

### Metagenomic Analysis of Illumina Genome Analyzer CPC98 Reads

10.64% of the 840,000 sequence reads analysed were classified as belonging to the infraorder *Pecora*, with over half (5.44%) specifically classified as derived from *B. taurus*. The next most highly represented species were human (*Homo sapiens*) (1.11% of reads) and mouse (*Mus musculus*, 0.76% of reads). 4.52% of reads were classified as derived from green plants, 4.08% from fungi, 0.87% from bacteria and 0.14% from viruses. The majority of reads were either not assigned to any taxon (33.04% unknown) or classified very poorly (*e.g.* 24.19% were classified only as belonging to the *Eukaryota*) (Figure S1).

## Discussion

### Aurochs mtDNA Sequence Authenticity and Preservation

Previous investigations have shown that the sequencing of ancient DNA is particularly sensitive to two major sources of error. Firstly, contamination with modern genetic material can result in the generation of inauthentic DNA sequences [33]. Secondly, chemical modification of the bases in ancient sequences, primarily the deamination of cytosine residues to uracil, can cause nucleotide misincorporation in newly synthesised DNA during PCR amplification or DNA sequencing [25], [44]. For the aurochs mtDNA genome presented here, four lines of evidence indicate that errors from both sources are low.

Firstly, we have achieved a high average sequence coverage of the CPC98 mtDNA genome using Illumina GA (9.1×) and Sanger (7.9× in total, with data generated from between two and seven independent primary multiplex PCRs) sequencing methodologies generated from multiple independent DNA extracts. For all nucleotide positions at which both Sanger and Illumina GA methods gave high-confidence calls, no discrepancies were observed. The consensus mtDNA sequence generated from both sequencing methods has a combined average sequencing depth of 16.9× across the complete genome (16,338 bp). This figure is comparable to the sequencing coverage generated for another ancient mammalian mtDNA genome recently retrieved from a bone sample [26].

Secondly, we see no evidence for contamination with modern bovine DNA. While our analysis does not exclude the possibility that the CPC98 mtDNA genome sequence has been contaminated with a modern haplogroup P sequence, this scenario seems unlikely given the extremely low frequency of this haplogroup in modern samples [6], [8], [14], [16], [17]. When mtDNA sequences from all other modern bovine haplogroups (I, R, Q and T) are considered, we obtain a maximum possible estimate of modern contamination of 0.85%. Although comparable to the levels of modern contamination reported for ancient Neanderthal complete mtDNA genomes [21], [26] this figure also corresponds to the observed rate of Illumina GA sequencing error detected for the CPC98 sample (discussed below).

We also observed that the number of Illumina GA reads mapping to the human genome, possibly indicating modern human contamination, is low. Of the total number of non-duplicated, non-Illumina GA adaptor sequence contaminant reads, 21.68% mapped exclusively to the bovine genome, compared with 0.52% mapping to both the bovine and human genomes. The latter group can most likely be explained by the presence of highly conserved sequences existing between the two genomes [38]. Also, 0.14% of reads mapped solely to the human genome and did not match any sequence in the current bovine genome assembly. While this may be due to modern human contamination, and thus provide an upper estimate of any such contamination, it may equally reflect the incomplete status of the bovine genome sequence [38].

Thirdly, we observed no excess of the characteristic C-to-T and G-to-A misincorporations due to *post-mortem* cytosine deamination historically observed in ancient DNA studies. The absence of such nucleotide lesions in the CPC98 mtDNA sequence is attributable to the use, during Illumina GA library preparation, of Phusion® High-Fidelity DNA polymerase enzyme (New England Biosciences, see Materials and Methods section), which does not amplify efficiently through deaminated products of cytosine [45]. The sequence errors observed are therefore not reflective of DNA diagenesis in the CPC98 but are most likely artefacts of the sequencing method used. We estimated the sequencing error rate for the individual Illumina GA reads to be 1.57%. Notably, the majority of errors occurred within the six 3′-most nucleotides of reads. This bias in the location of mismatched nucleotides, together with the observed excess of C-to-A and G-to-T sequencing errors, has previously been described as being inherent to the Illumina GA sequencing technology [46].

The proportion of non-duplicate, non-adaptor Illumina GA reads mapping to the aurochs (21.68%) is relatively high when compared with estimates of endogenous genetic material from other palaeogenomics surveys. While this figure is lower than that reported for ancient material retrieved from permafrost-preserved mammoth samples [32], [47], it is substantially higher when compared to other palaeogenetic studies where the proportion of endogenous sequences was estimated at between ∼0.3%–6.0% [48], [49], [50], [51].

Finally, metagenomic analyses of the Illumina GA reads showed a high percentage of unambiguous *B. taurus* sequences. A substantially larger number of reads were classified as being derived from the infraorder *Pecora* with *Bos* spp. being the most probable source. Although this analysis identified a number of potential human sequences, we believe that this is due to over-representation of human genomic sequence in the GenBank non-redundant nucleotide database rather than contamination with modern human DNA. In support of this, a comparable number of Illumina GA reads were classified as *M. musculus*, another highly represented species in the database. It is difficult to make further conclusions regarding the other species contributing to the metagenome of CPC98 given the large number of unidentified sequences, reflecting the short Illumina GA read length used in this experiment [52].

Collectively, these findings support the contention that the *B. primigenius* mtDNA genome presented here is authentic.

### Heteroplasmy in an Ancient mtDNA Genome

Our analysis revealed the presence of a single heteroplasmic nucleotide position (16,121) in the CPC98 mtDNA genome sequence. A recent assessment of complete human mtDNA genome sequences showed heteroplasmy to be relatively common, occurring in ∼6.0% of all analysed samples [53]. The heteroplasmic nucleotide position detected here occurs in the hypervariable region of the control region and has been previously identified as being a site where multiple substitutions have occurred [8]. Consequently, our observation of heteroplasmy is highly plausible.

In this study, heteroplasmy in CPC98 was identified first via Illumina GA sequencing and then later confirmed by analysis of the Sanger chromatograms. The high sequence depth, combined with the digital nature of the NGS data, enables secure identification of heteroplasmic nucleotide loci in a way not previously possible given the analog nature of Sanger chromatogram data. Heteroplasmy has also been recently detected in an analysis of mtDNA sequences generated from archaeological human remains [24], and at a variable number tandem repeat (VNTR) locus in a complete mammoth mtDNA genome [54].

### Genetic History of European *B. primigenius* Haplogroup P

Previous investigations have demonstrated that the predominant haplogroup within the Eurasian aurochs was haplogroup P [8], [13]. Analysing the most variable portion of the mtDNA control region, Edwards et al. [13] showed that haplogroup P divergence predates divergence within macro-haplogroup T, the predominant haplogroup in modern *B. taurus* [7], [8], [13], [14], [17], [39]. The analysis of complete mtDNA genome sequences presented here corroborates these findings. Mean mtDNA coding region pairwise nucleotide diversity (π) for the two haplogroup P sequences is ∼1.2-fold higher than that for macro-haplogroup T (Table 3). The estimated divergence date for the two haplogroup P sequences (22,401 yBP) is 1.3-fold higher than that for macro-haplogroup T (16,000 yBP as estimated by Achilli et al. [16]). This divergence time agrees with the estimated time to the most recent common ancestor (TMRCA) for 51 predominantly Neolithic ancient haplogroup P samples of 17,230 years (95% confidence interval: 10,050 to 30,230 years) [13]. Our estimate gives the date of divergence relative to present day, whereas that of Edwards et al. [13] is relative to the deposition date of the ancient samples, on average 6,262 years ago. This difference in time reference points accounts for the numerical difference between the time estimates.

The reduced level of diversity in macro-haplogroup T compared to that in haplogroup P reflects the differences in demographic history between the groups. Macro-haplogroup T underwent a Neolithic genetic bottleneck and subsequent rapid expansion as a consequence of domestication and ensuing millennia of animal husbandry [3], [6], [8], [13], [16]. In contrast, the wild haplogroup P population existing in Europe until the arrival of macro-haplogroup T from the Near East was not subject to the same bottlenecking [8], [13].

### Prospects for a Complete *B. primigenius* Nuclear Genome

The excellent preservation of the aurochs DNA within the CPC98 humerus sample, as demonstrated by the analysis of the mtDNA genome, paves the way for the sequencing of a complete aurochs nuclear genome. This potential is further supported by the generation of a large number (∼7.9 million) of CPC98-derived DNA sequence reads (via Illumina GA sequencing) which map to regions of the *B. taurus* genome other than the mtDNA genome. Indeed, the mtDNA sequence presented here can be used during whole genome sequence assembly as an analytical standard of modern DNA contamination. This will provide confidence when obtaining multi-fold sequencing coverage of the complete aurochs genome.

Analysis of a complete *B. primigenius* genome sequence offers intriguing opportunities to study the recent evolutionary history of domesticated cattle and their wild ancestors, as well as providing insight into human prehistory. More crucially, a complete aurochs genome sequence will permit the identification of functional genetic differences between the *B. taurus* and *B. primigenius* lineages, especially those pertaining to agro-economic production traits that have been under intense selection, since the initial founding of domestic herds some 10,000 years ago.

## Materials and Methods

### Archaeological Sample Details

The aurochs sample analysed in this study (laboratory code CPC98) consists of the proximal half of a humerus retrieved from Carsington Pasture Cave, Derbyshire, England. (http://capra.group.shef.ac.uk/1/carsing.html). The control region of the mtDNA of this sample has been studied previously by Troy et al. [8] and Edwards et al. [13] using overlapping fragments of 201 bp and 411 bp, respectively. This sample was radiocarbon dated, as part of the analysis undertaken by Troy et al. [8] to 5,936±34 yBP (uncalibrated radiocarbon age). Using the online calibration programme CALIB (http://143.117.32.11/calib/), this equates to 6,738±68 cal. yBP. Therefore, this bone dates to the Mesolithic period in Britain and thus must be a *B. primigenius* animal, and not a morphological misidentification of a *B. taurus* animal. The Neolithic, and consequently domestic cattle, did not arrive in Britain until about 4,000 cal. BC [35]—a timeframe equivalent to approx. 6,000 cal. yBP. The thermal age of this bone, defined as the time taken to produce a given degree of DNA degradation when temperature is held at a constant 10°C [55], [56], was calculated at 6,999 years, using the online DNA Recovery Rate Calculator (DRRC; personal communication, D. Harker 2009). The pre-Neolithic radiocarbon date, along with the excellent preservation of the specimen as demonstrated by us in previous studies [8], [13], [14], were the primary reasons why it was chosen for both Sanger and Illumina GA sequencing.

### Ancient DNA Extraction

All ancient DNA extractions were performed in a specifically-dedicated ancient DNA laboratory. Powdered bone samples, weighing between 200 mg and 500 mg, were prepared using a small-scale version of the modified Yang et al. procedure [57] previously described by our group [8], [58], [59] except that 200 µg/ml proteinase K (rather than 100 µg/ml) was added to the extraction buffer. Additionally, 5 µg/ml phage λ carrier DNA was added to the extraction buffer for those extracts analysed via Sanger sequencing only; no λ carrier DNA was added to the extracts analysed via Illumina GA technologies. In total, 13 DNA extractions were performed and all extracts gave sequence products. 10 extractions were sequenced via the Sanger sequencing method, while the other three were used to make three separate Illumina GA libraries. No extractions were analysed with both methods.

### Sanger-Based DNA Sequencing of Ancient Aurochs DNA

#### Primer design for Sanger DNA sequencing

The complete *B. taurus* mitochondrial genome [36] (GenBank accession no. V00654) was aligned with the complete mitochondrial sequences of sheep (*Ovis aries* [60]) and pig (*Sus scrofa* [61]) to identify regions of the genome that are strongly conserved between species. Primers were designed in these regions to give 31 overlapping primer pairs covering the entire genome (Table S1). The average PCR product length was 646 bp (ranging between 471 bp and 701 bp) and the average overlap between products, excluding primers, was 81 bases (ranging between 15 bp and 251 bp).

#### Multiplex amplification of the complete aurochs mtDNA genome

Polymerase chain reaction (PCR) set-up was conducted in a laboratory dedicated solely to pre-amplification ancient work. As extracted DNA can be a limiting factor to amplifying a whole mtDNA genome, a modified version of the multiplex PCR approach [37] was carried out. In their paper, Krause et al. [37] developed the multiplex approach to amplify the entire mitochondrial genome of a mammoth from just two initial PCR amplifications. In brief, this was accomplished by using primer pairs covering overlapping DNA sequence fragments across the complete mitochondrial genome. These primer pairs were then combined into three sets, each containing every third primer pair, with either 10 or 11 primer pairs in each set (Table S2). This was to avoid amplification of the short overlapping fragments during the multiplex step. Each of the three sets was used in a multiplex PCR amplification that required the same amount of ancient DNA template as would usually be used for amplifying a single target sequence. Subsequently, the three primary amplifications were diluted and used as templates in secondary PCR reactions, in which each product was amplified individually.

Multiplex primer mixes were made by adding equal amounts of all primers in that set at a concentration of 100 µM, and then adding either 4.0 µl (Amplification Sets 1 and 3) or 4.4 µl (Amplification Set 2) to each 20 µl 1st-round PCR tube, to give a final concentration of 1 µM. PCR amplifications were in 20 µl reaction volumes containing the following: 1× PCR buffer (10 mM Tris•HCl [pH 8.8]; 50 mM KCl; 0.1% Triton X-100), 4.0 mM MgCl_2_, 200 µM dNTPs, 1.0 µM of the primer mix, 0.5 units of Platinum *Taq* polymerase (Invitrogen), and 7.5 µl DNA, equating to ∼15 mg of starting bone powder, as suggested by Krause et al. [37]. First-round multiplex PCR was carried out in a dedicated PCR thermal cycler. Cycling parameters were as follows: 2 min denaturation step at 94°C, followed by 27 cycles of 20 s denaturation at 94°C, 30 s of annealing at 52°C, and 60 s of extension at 72°C, followed by a final 4 min extension step at 72°C. Between two and seven independent primary PCRs were performed for each of the three multiplex mixes.

A second-round PCR was then performed in the main analytical laboratory. The products from each first-round multiplex PCR were diluted 40-fold (780 µl of ddH_2_O was added to each 20 µl PCR amplification). Ten or 11 separate reactions (depending on the amplification set; Table S2) were performed in duplicate from each multiplex PCR, with one of the 31 primer pairs added. Amplification conditions were as for the first-round PCR, except the final primer concentration was increased to 1.5 µM. Reactions took place with 12.5 µl of diluted first-round PCR added to 50 µl reaction volumes. PCR cycling conditions were the same as in the first-round, except 33 cycles were used instead of 27. Products were then visualized on 1.5% agarose mini-gels. Multiple extraction and PCR blanks were included for each amplification and remained negative throughout.

PCR products were cleaned using the QIAquick PCR Purification Kit (Qiagen) according to the manufacturer's instructions, but with an additional wash step and elution in 40 µl of 1× TE buffer. Five separate batches of purified PCR products were Sanger-sequenced commercially by Macrogen Inc. (http://www.macrogen.com). Numbers of amplicons generated per primer pair are shown in Table S3. GC content of the amplified products ranged from 35% to 58%, with an average of 43%.

#### Authentication of Sanger-sequenced whole mtDNA genome

The criteria for authenticating the mitochondrial genome sequence were as follows. Between two and five independent extractions were amplified for each primer region (Table S3). Reproducible data were designated as those that gave consistent sequences in at least four or more amplifications (numbers amplified per primer pair shown in Table S3). In order to be considered authentic, any mutations observed had to be replicated in sequences from two separate extracts. The Sanger mtDNA genome sequence derived from the CPC98 sample was, therefore, verified through independent extractions, amplifications and sequence determinations.

Both inter-lab and intra-lab replication was undertaken on separate samples of bone. Inter-lab replication was performed by B.S. at the Henry Wellcome Ancient Biomolecules Centre in Oxford. Using different extraction methods and primer pairs [62] a region of 654 bp was sequenced across the origin (from position 15,759 to 73), covering the tRNA-Pro gene and 68% of the 5′-end of the control region. Intra-lab replication was also carried at Trinity College Dublin as part of a *CYTB* study of aurochs samples [14]. Again, different primer pairs were used to amplify two non-over-lapping fragments from the *CYTB* gene, totalling 365 bp (nucleotide positions 14,673-14,900 and 15,031–15,167).

In ancient mtDNA analysis, sequences can be recovered that are not authentic but derive from some external contaminant or the nuclear genome. We regard our Sanger *B. primigenius* sequence as genuine for the following reasons. By using separate samples of bone, a subset of sequences was independently replicated by B.S. at the Henry Wellcome Ancient Biomolecules Centre in Oxford using different extraction methods and primer pairs [62]. The mtDNA sequence of the aurochs specimen is clearly of bovine origin but is unique. Each sequence generated using the Sanger method had a perfect match to overlapping fragments.

### Preparation of Ancient Aurochs DNA Extracts for Illumina Genome Analyzer Sequencing

Three independent extracts of aurochs DNA from sample CPC98 (labelled C1, C2 and C3), each organised into three separate ∼30 µl aliquots (labelled C1_1–3_, C2_1–3_ and C3_1–3_ —giving a total of nine aliquots), were prepared for high-throughput sequencing using an Illumina Genome Analyzer, in a dedicated ancient DNA facility based in the Smurfit Institute of Genetics, Trinity College Dublin. All 30 µl from each aurochs DNA extract aliquot (*n* = 9) was prepared for Illumina GA single-read DNA sequencing according to the Illumina Genome Analyzer Genomic DNA sample preparation kit protocol (Illumina, Catalogue no. 1003806). The step-wise details of the Illumina GA library preparations are detailed below.

#### Blunt end-repair of aurochs DNA extracts

Each 30 µl aurochs DNA extract was included in a 100 µl final reaction mixture containing 1× T4 DNA ligase buffer with 1 mM dATP (New England BioLabs [NEB]), 400 µM of each dNTP (Invitrogen), 15 units T4 DNA polymerase (NEB), 5 units DNA Polymerase I Large (Klenow) Fragment (NEB) and 50 units T4 polynucleotide kinase (NEB). Reaction mixtures were incubated at 20°C for 30 mins, after which end-repaired DNA was purified using a QIAquick PCR Purification Kit (Qiagen) and eluted in 32 µl of elution buffer according to manufacturer's instructions.

#### Creation of a single 3′-dATP overhang on the end-repaired aurochs DNA extracts

To facilitate Illumina GA adaptor ligation, a single ‘A’ base was added to the 3′-ends of the blunt-end repaired aurochs DNA extracts. 32 µl of purified phosphorylated blunt end-repaired aurochs extract DNA was included in a final 50 µl reaction mixture containing: 1× Klenow fragment buffer (NEB), 200 µM dATP (Invitrogen), and 15 units Klenow fragment with 3′-to-5′ exonuclease activity (NEB). Reactions were incubated at 37°C for 30 min, after which DNA was purified using a QIAquick MinElute Kit (Qiagen) and eluted in 19 µl of elution buffer according to manufacturer's instructions.

#### Illumina Genome Analyzer adaptor ligation

Ligation reactions (in 50 µl volumes) involved incubation of 19 µl of phosphorylated blunt-ended aurochs DNA extracts, with a 3′-dATP overhang, with 1× DNA ligase buffer (NEB), 1 µl of the proprietary Illumina GA single-read genomic adaptors (Illumina, catalogue no. FC-102-1003) and 10 units T4 DNA ligase (Invitrogen). Extracts were incubated at room temperature for 15 minutes, purified using QIAquick MinElute Kit (Qiagen) and eluted in 19 µl of elution buffer according to manufacturer's instructions.

#### Illumina Genome Analyzer DNA library preparations via PCR enrichment of purified end-repaired, adaptor-ligated DNA templates

Individual Illumina GA libraries (*n* = 9) were produced via PCR enrichment of the end-repaired adaptor-ligated DNA templates prior to sequencing. PCR amplifications (50 µl) comprised 19 µl of end-repaired-linker-ligated aurochs DNA, 1× Phusion® High-Fidelity DNA polymerase buffer (NEB), 1 µl of forward primer, 1 µl of reverse primer (Illumina, catalogue no. FC-102-1003), 250 nM of each dNTP (Invitrogen) and 1 unit Phusion® High-Fidelity DNA polymerase (NEB). PCR amplification reactions consisted of an initial denaturation step of 98°C for 30 s, 18 cycles of 98°C for 10 s, 65°C for 30 s and 72°C for 30 s, followed by a final extension step of 72°C for 5 min. PCR products were visualised following electrophoresis on a 1.5% agarose gel stained with ethidium bromide (0.5 µg/ml). All aurochs DNA extract aliquots yielded successful genomic libraries for Illumina GA sequencing. Examination of the PCR products indicated the majority of the aurochs DNA inserts within the Illumina GA libraries were ∼40–60 bp in length. Individual libraries (*n* = 9) were subsequently pooled according to their initial extract number (C1_1–3_, C2_1–3_ and C3_1–3_) to form three final libraries labelled C1, C2 and C3, respectively. Pooled libraries were purified using a QIAquick PCR Purification Kit (Qiagen) and eluted in 30 µl elution buffer according to manufacturer's instructions. Purified libraries were quantified using a Qubit™ fluorometer (Invitrogen) and a Quant-iT™ double-stranded DNA High-Sensitivity Assay Kit (Invitrogen). The final molar concentration of each of the three pooled libraries ranged between 0.51–0.66 µM.

#### Illumina Genome Analyzer DNA sequencing

Cluster generation and sequencing were carried out on an Illumina cluster station and Genome Analyzer II sequencer according to the manufacturer's instructions. Libraries were sequenced as single read 36-mers using the standard Illumina Genome Analyzer pipeline. Intensity files generated by the IPAR server software were base called using Bustard (the Illumina base caller). The first flow cell was processed using pipeline version 1.0. All subsequent flow cells were processed using pipeline version 1.3. The Illumina GA-generated FASTQ quality scores from pipeline 1.0 and pipeline 1.3 were converted to PHRED scores (Sanger encoded) for use with MAQ [63].

### Assembly of a *B. primigenius* mtDNA Genome from Illumina Genome Analyzer Sequence Reads

#### Template sequences

The bovine genome (version 4.0, released October 2007) sequences for all chromosomes and unmapped scaffolds were downloaded from the UCSC genome browser website (http://genome.ucsc.edu). The GenBank haplogroup P complete mtDNA genome sequence (GenBank accession no. DQ124389) was downloaded in place of the Hereford bovine genome 4.0 mtDNA sequence. The human genome sequence (hg19/GRCh37, released February 2009) was also downloaded.

#### Alignment of Illumina Genome Analyzer reads to genome sequences

Illumina GA reads were aligned to the bovine and human genome sequences using the software package MAQ (Mapping Alignment with Quality, available from http://maq.sourceforge.net). Prior to alignment, contaminant sequence reads exactly matching the 33bp Illumina GA adaptor sequence were removed using the MAQ program. Additional reads matching at least 12 out of any 13 consecutive adaptor nucleotides were excluded from the analyses. The MAQ program was used to align the remaining sequence reads with the genome template, permitting up to three mismatches per 36-nucleotide read. Aligned sequences mapping to the DQ124389 bovine mtDNA genome sequence were assembled into a consensus sequence with associated quality information for each nucleotide position using the ‘assemble’ option of MAQ. Data on the mapping of individual reads was obtained using the ‘pileup’ option of MAQ.

#### Metagenomic analyses of Illumina Genome Analyzer reads

Metagenomic analyses was performed to produce a taxonomic profile of the of the Illumina GA CPC98 reads. For this, 60,000 non-adaptor reads were selected randomly from each of 14 flow-cell lanes and BLAST searched against the GenBank non-redundant nucleotide database (downloaded November 2^nd^ 2009) using the BLASTN program [64] with a word size of 7 and an expectation value of 100. Metagenomic analysis was performed on the BLAST output using the software application MEGAN [52] to determine the lowest common taxonomic ancestor for each sequence read.

### mtDNA Sequence Analysis and Phylogenetics

Full length *Bos* spp. mtDNA sequences were obtained from GenBank. Sequences were aligned manually using MEGA version 4 (Molecular Evolutionary Genetic Analysis [65], Table S4). Neighbour joining trees were constructed in MEGA 4 using the maximum composite likelihood method, with all positions containing gaps and missing data eliminated from the dataset. 1,000 bootstrap replicates were performed for each tree. The TMRCA for haplogroup P and 95% confidence interval for this time estimate was calculated using the program CRED [66]. Diversity statistics were calculated using Arlequin Version 2.0 [67]. The positions of the gene and control region sequences within the mtDNA genome are detailed in Table S5.

## Supporting Information

Figure S1 MEGAN metagenomic analysis of the aurochs CPC98 Illumina GA reads. 60,000 non-adapter reads were selected randomly from each of 14 flow-cell lanes and BLAST searched against the GenBank non-redundant nucleotide database. Results were analysed using MEGAN software. Figures shown are percentages of the total 840,000 reads assigned to different taxa.
(0.60 MB TIF)

Table S1 PCR primer sequence information for each of the 31 overlapping amplicons used for Sanger sequencing. Nucleotide positions are given based on the reference bovine mtDNA genome sequence (GenBank accession no. V00654).
(0.07 MB DOC)

Table S2 A list of the multiplex PCR amplicon sets.
(0.03 MB DOC)

Table S3 The number of PCR amplicons generated per primer pair.
(0.06 MB DOC)

Table S4 A list of all complete bovine mtDNA sequences used for sequence and phylogenetic analysis in this study. The GenBank accession number and the macro-haplogroup/haplogroup to which individual mtDNA genome sequence belongs are provided.
(0.15 MB DOC)

Table S5 The nucleotide positions of each mtDNA gene and the mtDNA control region. Base positions are according to the *Bos taurus* reference mtDNA genome sequence (GenBank accession no. V00654).
(0.07 MB DOC)
