# Supplementary figures and images for: A Complete Mitochondrial Genome Sequence from a Mesolithic Wild Aurochs (Bos primigenius)
Source: PLoS One. 2010 Feb 17;5(2):e9255. doi: 10.1371/journal.pone.0009255 (PMC2822870; doi:10.1371/journal.pone.0009255)

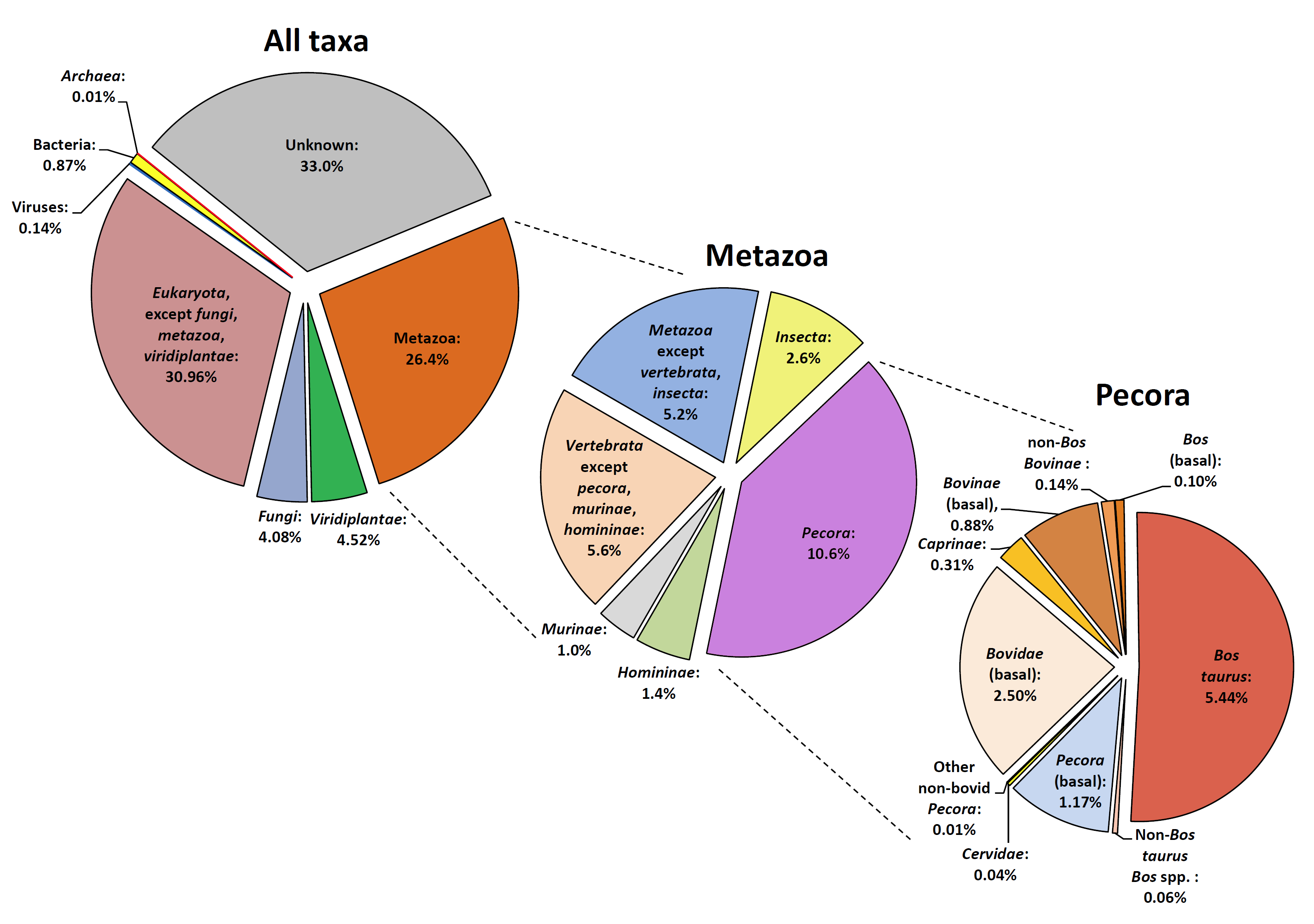

Supplement: Figure S1 — MEGAN metagenomic analysis of the aurochs CPC98 Illumina GA reads. 60,000 non-adapter reads were selected randomly from each of 14 flow-cell lanes and BLAST searched against the GenBank non-redundant nucleotide database. Results were analysed using MEGAN software. Figures shown are percentages of the total 840,000 reads assigned to different taxa. (0.60 MB TIF) [file pone.0009255.s001.tif]
