# Supplementary material for: A Complete Mitochondrial Genome Sequence from a Mesolithic Wild Aurochs (Bos primigenius)
Source: PLoS One. 2010 Feb 17;5(2):e9255. doi: 10.1371/journal.pone.0009255 (PMC2822870; doi:10.1371/journal.pone.0009255)
Supplement: Table S1 — PCR primer sequence information for each of the 31 overlapping amplicons used for Sanger sequencing. Nucleotide positions are given based on the reference bovine mtDNA genome sequence (GenBank accession no. V00654). (0.07 MB DOC) [file pone.0009255.s002.doc]

**Table S1.** PCR primer sequence information for each of the 31 overlapping amplicons used for Sanger sequencing.

| Forward primer details | | | | Reverse primer details | | | | PCR amplicon length (bp) | PCR amplicon set |
| --- | --- | --- | --- | --- | --- | --- | --- | --- | --- |
| Primer name | Sequence (5’-3’) | Start position | Finish position | Primer name | Sequence (5’-3’) | Start position | Finish position |
| P1FOR | ATCCTTCTAGAGGAGCCTGTT | 964 | 984 | P1REV | GGTACAAGGGGTAATCTTTGC | 1637 | 1617 | 674 | 1 |
| P2FOR | GTACCGCAAGGGAACGATGAA | 1571 | 1591 | P2REV | ATCTTTCCTTAGATGCACTCC | 2249 | 2229 | 679 | 2 |
| P3FOR | GTGCCTGATAATACTCTGACC | 2143 | 2163 | P3REV | AACGAACCTTGATAGCGGTT | 2832 | 2812 | 690 | 3 |
| P4FOR | CGACCTCGATGTTGGATCAGG | 2776 | 2796 | P4REV | GAATGGAGTATACGGCTAGGC | 3449 | 3429 | 674 | 1 |
| P5FOR | TATCATAGCTTTAGGCCTAGC | 3325 | 3345 | P5REV | ATGCTCGGATTCATAGGAAGG | 3926 | 3906 | 602 | 2 |
| P6FOR | CACAATCCACACATACCAGAA | 3839 | 3859 | P6REV | TATTACGGTTCATTGGCC | 4526 | 4509 | 688 | 3 |
| P7FOR | ACAACCCACGAGCTACAGAA | 4408 | 4427 | P7REV | GAAAGTGGGTAGAATGATGC | 5108 | 5089 | 701 | 1 |
| P8FOR | ACTCTCCTATCCATAGG | 5004 | 5020 | P8REV | ATGGCTGAGCAAAGCATT | 5679 | 5662 | 676 | 2 |
| P9FOR | AAGCTGCTTCTCTGAATTTGC | 5568 | 5588 | P9REV | TACGGATCATACGAACAGAG | 6250 | 6231 | 683 | 3 |
| P10FOR | CCTCAATTTTAGGAGCCATCAA | 6153 | 6174 | P10REV | CGTAGTATGTGTCGTGAA | 6803 | 6786 | 651 | 1 |
| P11FOR | GGCAACACTTCATGGAGGTAA | 6658 | 6678 | P11REV | TTGAGAGAGACATAGAGG | 7299 | 7282 | 642 | 2 |
| P12FOR | AGAACCCACCTATGTTAACC | 7204 | 7223 | P12REV | TCAGAGGAGACTAACATTCG | 7843 | 7824 | 640 | 3 |
| P13FOR | GGAGCTACGACTATTAGAA | 7766 | 7784 | P13REV | GTTGGAGGGTTACAAAGCG | 8428 | 8410 | 663 | 1 |
| P14FOR | CCTTATCGTACTATTCCCAAGC | 8352 | 8373 | P14REV | AGCTCCTGTAAGAGGTCAAGG | 9032 | 9012 | 681 | 2 |
| P15FOR | GACAACACATAATGACACACC | 8959 | 8979 | P15REV | AGACAATTAAGAAGGTGGACC | 9621 | 9601 | 663 | 3 |
| P16FOR | CTCAGAATACTATGAAGCACC | 9503 | 9523 | P16REV | GTTCACTCATAGGCTAGGCTT | 10142 | 10122 | 640 | 1 |
| P17FOR | CCACTGCCATGAGCCTCACAA | 10042 | 10062 | P17REV | GTGGAGTGGATAGGGAGTCGG | 10721 | 10701 | 680 | 2 |
| P18FOR | TTAGCTTTACAAGCCTCCTCC | 10629 | 10649 | P18REV | GGAGAGTATAATGAATGGGTATGC | 11323 | 11300 | 695 | 3 |
| P19aFOR | TACAGTACTGAGTACAACC | 11064 | 11082 | P19aREV | TTATGGTTCGGCTGTGGATTCGTT | 11555 | 11532 | 492 | 1 |
| P19bFOR | TCCTTATCCAGACACCTTGAAG | 11430 | 11451 | P19bREV | GGGAGCATAGAATTAGCAGTT | 12012 | 11992 | 583 | 2 |
| P20FOR | GGAGTAAATATAGTAATCACCGCC | 11717 | 11740 | P20REV | AGATCATGTGACGAATAGTGC | 12405 | 12385 | 689 | 3 |
| P21FOR | TCAAACTGACACTGACTAACC | 12298 | 12318 | P21REV | AATAGTGTGGTAATGGCTCC | 12968 | 12949 | 671 | 1 |
| P22FOR | AGGTATCTTCCTACTAATCCG | 12873 | 12893 | P22REV | ATGTATCCTGCGAAGAGGCTT | 13508 | 13488 | 636 | 2 |
| P23FOR | TTCGCACTTCTAGGACAA | 13393 | 13410 | P23REV | GGGTGATTTATGATACAGG | 14067 | 14049 | 675 | 3 |
| P24FOR | ACTGTATAAAGCCGCAATCC | 13997 | 14016 | P24REV | GTGTATTGCTAGGAATAGGCC | 14675 | 14655 | 679 | 1 |
| P25FOR | TGCATTCATCGACCTTCCAGC | 14561 | 14581 | P25REV | GTCCTTAATGGTATAGTAGG | 15197 | 15178 | 637 | 2 |
| P26FOR | CCACGAAACAGGCTCCAACAA | 15113 | 15134 | P26REV | CTTCCTTGAGTCTTAGGGAGG | 15734 | 15714 | 622 | 3 |
| P27aFOR | TATTCCGACCACTCAGCCAA | 15460 | 15479 | P27aREV | GTGTATGGGCGTGTTATGTT | 15930 | 15911 | 471 | 1 |
| P27bFOR | CTGGTCTTGTAAACCAGAGAA | 15680 | 15700 | P27bREV | GAAAGAACCAGATGCCT | 16278 | 16262 | 599 | 2 |
| P28FOR | ATTACCATGCCGCGTGAA | 16163 | 16180 | P28REV | TAACAGGAAGGCTGGGACCAA | 458 | 438 | 535 | 3 |
| P29FOR | CCGTTGATGTAGCTTAA | 362 | 378 | P29REV | AGGGTTTGCTGAAGATGG | 1061 | 1044 | 700 | 2 |
