# Supplementary material for: A Complete Mitochondrial Genome Sequence from a Mesolithic Wild Aurochs (Bos primigenius)
Source: PLoS One. 2010 Feb 17;5(2):e9255. doi: 10.1371/journal.pone.0009255 (PMC2822870; doi:10.1371/journal.pone.0009255)
Supplement: Table S2 — A list of the multiplex PCR amplicon sets. (0.03 MB DOC) [file pone.0009255.s003.doc]

**Table S2.** A list of the multiplex PCR amplicon sets

| **Amplification set 1** | **Amplification set 2** | **Amplification set 3** |
| --- | --- | --- |
| 1 | 2 | 3 |
| 4 | 5 | 6 |
| 7 | 8 | 9 |
| 10 | 11 | 12 |
| 13 | 14 | 15 |
| 16 | 17 | 18 |
| 19a | 19b | 20 |
| 21 | 22 | 23 |
| 24 | 25 | 26 |
| 27a | 27b | 28 |
|  | 29 |  |
| 10 primer sets | 11 primer sets | 10 primer sets |
