# Supplementary material for: A Complete Mitochondrial Genome Sequence from a Mesolithic Wild Aurochs (Bos primigenius)
Source: PLoS One. 2010 Feb 17;5(2):e9255. doi: 10.1371/journal.pone.0009255 (PMC2822870; doi:10.1371/journal.pone.0009255)
Supplement: Table S3 — The number of PCR amplicons generated per primer pair. (0.06 MB DOC) [file pone.0009255.s004.doc]

**Table S3.** The number of PCR amplicons generated per primer pair

| **Primer set** | **Extraction #** | **Total seqs generated** | **Sequencing runs** | **Useable seq length (bp)** |
| --- | --- | --- | --- | --- |
| **P1** | 2 | 6 | 2 | 634 |
| **P2** | 3 | 7 | 2 | 639 |
| **P3** | 2 | 8 | 2 | 650 |
| **P4** | 6 | 17 | 4 | 634 |
| **P5** | 2 | 8 | 2 | 562 |
| **P6** | 2 | 8 | 2 | 651 |
| **P7** | 2 | 4 | 2 | 663 |
| **P8** | 4 | 8 | 2 | 643 |
| **P9** | 3 | 10 | 2 | 644 |
| **P10** | 2 | 5 | 2 | 613 |
| **P11** | 2 | 8 | 2 | 605 |
| **P12** | 2 | 8 | 2 | 602 |
| **P13** | 4 | 8 | 2 | 627 |
| **P14** | 4 | 7 | 3 | 640 |
| **P15** | 4 | 11 | 3 | 623 |
| **P16** | 2 | 8 | 2 | 600 |
| **P17** | 2 | 8 | 2 | 640 |
| **P18** | 3 | 8 | 2 | 652 |
| **P19a** | 5 | 8 | 3 | 451 |
| **P19b** | 2 | 5 | 2 | 542 |
| **P20** | 2 | 8 | 2 | 646 |
| **P21** | 4 | 10 | 2 | 632 |
| **P22** | 2 | 5 | 2 | 596 |
| **P23** | 2 | 10 | 2 | 640 |
| **P24** | 7 | 13 | 3 | 640 |
| **P25** | 2 | 4 | 2 | 598 |
| **P26** | 2 | 8 | 2 | 581 |
| **P27a** | 3 | 7 | 2 | 433 |
| **P27b** | 4 | 8 | 2 | 563 |
| **P28** | 2 | 4 | 2 | 497 |
| **P29** | 2 | 6 | 2 | 667 |
