# Supplementary material for: A Complete Mitochondrial Genome Sequence from a Mesolithic Wild Aurochs (Bos primigenius)
Source: PLoS One. 2010 Feb 17;5(2):e9255. doi: 10.1371/journal.pone.0009255 (PMC2822870; doi:10.1371/journal.pone.0009255)
Supplement: Table S4 — A list of all complete bovine mtDNA sequences used for sequence and phylogenetic analysis in this study. The GenBank accession number and the macro-haplogroup/haplogroup to which individual mtDNA genome sequence belongs are provided. (0.15 MB DOC) [file pone.0009255.s005.doc]

**Table S4.** A list of all complete bovine mtDNA sequences used for sequence and phylogenetic analysis in this study.

| **Sample** | **GenBank Accession Number** | **Sample number (as per Achilli et al. 2009 [16])** | **Haplogroup** |
| --- | --- | --- | --- |
| *Bos taurus*_reference (V00654) | V00654 | BRSa | T3 |
| UK_aurochs_CPC98 |  |  | P |
| UCSC_ref_mtdna_Hereford |  |  | T3a-T4 |
| Genbank_DQ124389.1 | DQ124389 | 100 | P |
| gi|237780775|gb|GQ129208.1|_Bos_tauru... | GQ129208 |  | T3a |
| gi|237780761|gb|GQ129207.1|_Bos_tauru... | GQ129207 |  | T3 |
| gi|229501214|gb|FJ971080.1|_Bos_tauru... | FJ971080 |  | Q |
| gi|229501228|gb|FJ971081.1|_Bos_tauru... | FJ971081 |  | Q |
| EU177867.1_isolate_Bos18 | EU177867 | 99 | Q1 |
| EU177866.1_isolate_Bos3 | EU177866 | 98 | Q1 |
| gi|229501242|gb|FJ971082.1|_Bos_tauru... | FJ971082 |  | Q1 |
| gi|229501256|gb|FJ971083.1|_Bos_tauru... | FJ971083 |  | Q1 |
| gi|229501270|gb|FJ971084.1|_Bos_tauru... | FJ971084 |  | R |
| gi|229501284|gb|FJ971085.1|_Bos_tauru... | FJ971085 |  | R |
| gi|229501298|gb|FJ971086.1|_Bos_tauru... | FJ971086 |  | R |
| gi|229501312|gb|FJ971087.1|_Bos_tauru... | FJ971087 |  | R |
| gi|229501326|gb|FJ971088.1|_Bos_tauru... | FJ971088 |  | I1 |
| gi|50234066|ref|NC_005971.1|_Bos_indi... | NC_005971 | 101 | I1 |
| gi|37545825|gb|AY126697.1|_Bos_indicu... | AY126697 |  | I1 |
| gi|33321647|gb|AF492350.1|_Bos_indicu... | AF492350 | 105 | I2 |
| EU177870.1_isolate_Bos49 | EU177870 | 104 | I2 |
| EU177869.1_isolate_Bos50 | EU177869 | 103 | I2 |
| EU177868.1_isolate_Bos51 | EU177868 | 102 | I1 |
| AF492351.1 | AF492351 |  | T3 |
| NC_006853.1 | NC_006853 |  | T4 |
| EU177865.1_isolate_Bos39 | EU177865 | 97 | T5 |
| EU177864.1_isolate_Bos38 | EU177864 | 96 | T5 |
| EU177863.1_isolate_Bos36 | EU177863 | 95 | T5 |
| EU177862.1_isolate_Bos35 | EU177862 | 94 | T5 |
| EU177861.1_isolate_Bos22 | EU177861 | 93 | T2 |
| EU177860.1_isolate_Bos15 | EU177860 | 92 | T2 |
| EU177859.1_isolate_Bos13 | EU177859 | 91 | T2 |
| EU177858.1_isolate_Bos12 | EU177858 | 90 | T2 |
| EU177857.1_isolate_Bos16 | EU177857 | 89 | T2 |
| EU177856.1_isolate_Bos41 | EU177856 | 88 | T2 |
| EU177855.1_isolate_Bos23 | EU177855 | 87 | T2 |
| EU177854.1_isolate_Bos8 | EU177854 | 86 | T2 |
| EU177853.1_isolate_Bos10 | EU177853 | 85 | T2 |
| EU177852.1_isolate_GR68 | EU177852 | 84 | T2 |
| EU177851.1_isolate_Bos21 | EU177851 | 83 | T2 |
| EU177850.1_isolate_Bos5 | EU177850 | 82 | T2 |
| EU177849.1_isolate_GR316 | EU177849 | 81 | T2 |
| EU177848.1_isolate_Bos37 | EU177848 | 77 | T1a |
| EU177847.1_isolate_Bos24 | EU177847 | 76 | T1a |
| EU177846.1_isolate_Bos17 | EU177846 | 75 | T1a |
| EU177845.1_isolate_Bos7 | EU177845 | 74 | T1a |
| EU177844.1_isolate_Bos9 | EU177844 | 73 | T1a |
| EU177843.1_isolate_Bos4 | EU177843 | 72 | T1a |
| EU177842.1_isolate_Bos2 | EU177842 | 71 | T1 |
| EU177841.1_isolate_Bos6 | EU177841 | 69 | T1 |
| EU177840.1_isolate_Bos19 | EU177840 | 68 | T1'2'3 |
| EU177839.1_isolate_Bos11 | EU177839 | 67 | T3 |
| EU177838.1_isolate_Bos14 | EU177838 | 66 | T3 |
| EU177837.1_isolate_Bos43 | EU177837 | 63 | T3 |
| EU177836.1_isolate_Bos42 | EU177836 | 62 | T3 |
| EU177835.1_isolate_Bos40 | EU177835 | 61 | T3 |
| EU177834.1_isolate_Bos54 | EU177834 | 60 | T3 |
| EU177833.1_isolate_Bos53 | EU177833 | 59 | T3 |
| EU177832.1_isolate_Bos52 | EU177832 | 58 | T3 |
| EU177831.1_isolate_Bos27 | EU177831 | 57 | T3 |
| EU177830.1_isolate_Bos20 | EU177830 | 56 | T3 |
| EU177829.1_isolate_Bos28 | EU177829 | 55 | T3 |
| EU177828.1_isolate_Bos48 | EU177828 | 53 | T3 |
| EU177827.1_isolate_Bos31 | EU177827 | 52 | T3 |
| EU177826.1_isolate_Bos30 | EU177826 | 51 | T3 |
| EU177825.1_isolate_Bos26 | EU177825 | 50 | T3 |
| EU177824.1_isolate_Bos25 | EU177824 | 49 | T3 |
| EU177823.1_isolate_Bos1 | EU177823 | 48 | T3 |
| EU177822.1_isolate_Bos29 | EU177822 | 47 | T3 |
| EU177821.1_isolate_Bos45 | EU177821 | 11 | T3 |
| EU177820.1_isolate_Bos47 | EU177820 | 6 | T3 |
| EU177819.1_isolate_Bos46 | EU177819 | 5 | T3 |
| EU177818.1_isolate_Bos34 | EU177818 | 4 | T3 |
| EU177817.1_isolate_Bos33 | EU177817 | 3 | T3 |
| EU177816.1_isolate_Bos44 | EU177816 | 2 | T3 |
| EU177815.1_isolate_Bos32 | EU177815 | 1 | T3 |
| DQ124418.1_isolate_H16 | DQ124418 | 27 | T3a |
| DQ124417.1_isolate_H15 | DQ124417 | 28 | T3a |
| DQ124416.1_isolate_H14 | DQ124416 | 19 | T3 |
| DQ124415.1_isolate_H13 | DQ124415 | 7 | T3 |
| DQ124414.1_isolate_H12 | DQ124414 | 13 | T3 |
| DQ124413.1_isolate_H11 | DQ124413 | 10 | T3 |
| DQ124412.1_isolate_H10 | DQ124412 | 26 | T4 |
| DQ124411.1_isolate_H9 | DQ124411 | 46 | T3 |
| DQ124410.1_isolate_H8 | DQ124410 | 17 | T3 |
| DQ124409.1_isolate_H7 | DQ124409 | 16 | T3 |
| DQ124408.1_isolate_H6 | DQ124408 | 9 | T3 |
| DQ124407.1_isolate_H5 | DQ124407 | 8 | T3 |
| DQ124406.1_isolate_H4 | DQ124406 | 12 | T3 |
| DQ124405.1_isolate_H3 | DQ124405 | 15 | T3 |
| DQ124404.1_isolate_H2 | DQ124404 | 14 | T3 |
| DQ124402.1_isolate_FC16 | DQ124402 | 42 | T3 |
| DQ124401.1_isolate_FC15 | DQ124401 | 25 | T4 |
| DQ124400.1_isolate_FC14 | DQ124400 | 24 | T4 |
| DQ124399.1_isolate_FC13 | DQ124399 | 70 | T1 |
| DQ124398.1_isolate_FC12 | DQ124398 | 40 | T3 |
| DQ124397.1_isolate_FC11 | DQ124397 | 33 | T3 |
| DQ124396.1_isolate_FC10 | DQ124396 | 80 | T2 |
| DQ124395.1_isolate_FC9 | DQ124395 | 44 | T3 |
| DQ124394.1_isolate_FC8 | DQ124394 | 39 | T3 |
| DQ124393.1_isolate_FC7 | DQ124393 | 79 | T2 |
| DQ124392.1_isolate_FC6 | DQ124392 | 23 | T4 |
| DQ124391.1_isolate_FC5 | DQ124391 | 32 | T3 |
| DQ124390.1_isolate_FC4 | DQ124390 | 45 | T3 |
| DQ124389.1_isolate_FC3 | DQ124389 | 100 | P |
| DQ124388.1_isolate_FC2 | DQ124388 | 38 | T3 |
| DQ124387.1_isolate_FC1 | DQ124387 | 43 | T3 |
| DQ124386.1_isolate_KC16 | DQ124386 | 65 | T3 |
| DQ124385.1_isolate_KC15 | DQ124385 | 18 | T3 |
| DQ124384.1_isolate_KC14 | DQ124384 | 54 | T3 |
| DQ124383.1_isolate_KC13 | DQ124383 | 78 | T2 |
| DQ124382.1_isolate_KC12 | DQ124382 | 41 | T3 |
| DQ124381.1_isolate_KC11 | DQ124381 | 37 | T3 |
| DQ124380.1_isolate_KC10 | DQ124380 | 36 | T3 |
| DQ124379.1_isolate_KC9 | DQ124379 | 34 | T3 |
| DQ124378.1_isolate_KC8 | DQ124378 | 31 | T3 |
| DQ124377.1_isolate_KC7 | DQ124377 | 22 | T4 |
| DQ124376.1_isolate_KC6 | DQ124376 | 64 | T3 |
| DQ124375.1_isolate_KC5 | DQ124375 | 21 | T4 |
| DQ124374.1_isolate_KC4 | DQ124374 | 35 | T3 |
| DQ124373.1_isolate_KC3 | DQ124373 | 30 | T3 |
| DQ124372.1_isolate_KC2 | DQ124372 | 20 | T4 |
| DQ124371.1_isolate_KC1 | DQ124371 | 29 | T3a |
| AY676873.1_isolate_32027 | AY676873 |  | T3 |
| AY676872.1_isolate_32026 | AY676872 |  | T3 |
| AY676871.1_isolate_32025 | AY676871 |  | T3 |
| AY676870.1_isolate_32024 | AY676870 |  | T1'2'3 |
| AY676869.1_isolate_32023 | AY676869 |  | T3 |
| AY676868.1_isolate_32022 | AY676868 |  | T3 |
| AY676867.1_isolate_32021 | AY676867 |  | T1'2'3 |
| AY676866.1_isolate_32020 | AY676866 |  | T3b |
| AY676865.1_isolate_32019 | AY676865 |  | T3b |
| AY676864.1_isolate_32018 | AY676864 |  | T3 |
| AY676863.1_isolate_32017 | AY676863 |  | T3b |
| AY676862.1_isolate_32016 | AY676862 |  | T3b |
| AY676861.1_isolate_26368 | AY676861 |  | T3 |
| AY676860.1_isolate_25651 | AY676860 |  | T3 |
| AY676859.1_isolate_23854 | AY676859 |  | T3 |
| AY676858.1_isolate_22130 | AY676858 |  | T3b |
| AY676857.1_isolate_18905 | AY676857 |  | T3 |
| AY676856.1_isolate_17605 | AY676856 |  | T2 |
| AY676855.1_isolate_16683 | AY676855 |  | T3 |
| AY526085.1 | AY526085 |  | T3a-T4 |
| AB074968.1_haplotype:JBC8 | AB074968 |  | T3 |
| AB074967.1_haplotype:JBC7 | AB074967 |  | T3 |
| AB074966.1_haplotype:JBC6 | AB074966 |  | T3 |
| AB074965.1_haplotype:JBC5 | AB074965 |  | T3 |
| AB074964.1_haplotype:JBC3 | AB074964 |  | T4 |
| AB074963.1_haplotype:JBC2 | AB074963 |  | T4 |
| AB074962.1_haplotype:JBC1 | AB074962 |  | T4 |
