# Supplementary material for: A Complete Mitochondrial Genome Sequence from a Mesolithic Wild Aurochs (Bos primigenius)
Source: PLoS One. 2010 Feb 17;5(2):e9255. doi: 10.1371/journal.pone.0009255 (PMC2822870; doi:10.1371/journal.pone.0009255)
Supplement: Table S5 — The nucleotide positions of each mtDNA gene and the mtDNA control region. Base positions are according to the Bos taurus reference mtDNA genome sequence (GenBank accession no. V00654). (0.07 MB DOC) [file pone.0009255.s006.doc]

**Table S5.** The nucleotide positions of each mtDNA gene and the mtDNA control region.

| **Start** | **End** | **Genes / Feature** |
| --- | --- | --- |
| 1 | 363 | D-loop |
| 364 | 430 | tRNA-Phe |
| 431 | 1385 | 12 S ribosomal RNA |
| 1386 | 1452 | tRNA-Val |
| 1453 | 3023 | 16 S ribosomal RNA |
| 3024 | 3098 | tRNA-Leu |
| 3099 | 3100 |  |
| 3101 | 4056 | ND1 |
| 4057 | 4057 | ND1, tRNA-Ile |
| 4058 | 4122 | tRNA-Ile |
| 4123 | 4125 | tRNA-Ile, tRNA-Gln |
| 4126 | 4194 | tRNA-Gln |
| 4195 | 4196 |  |
| 4197 | 4265 | tRNA-Met |
| 4266 | 5307 | ND2 |
| 5308 | 5309 | ND2, tRNA-Trp |
| 5310 | 5374 | tRNA-Trp |
| 5375 | 5375 |  |
| 5376 | 5444 | tRNA-Ala |
| 5445 | 5445 |  |
| 5446 | 5518 | tRNA-Asn |
| 5519 | 5550 |  |
| 5551 | 5617 | tRNA-Cys |
| 5618 | 5685 | tRNA-Tyr |
| 5686 | 5686 |  |
| 5687 | 7228 | COX1 |
| 7229 | 7231 | COX1, tRNA-Ser |
| 7232 | 7299 | tRNA-Ser |
| 7300 | 7304 |  |
| 7305 | 7372 | tRNA-Asp |
| 7373 | 7373 |  |
| 7374 | 8057 | COX2 |
| 8058 | 8060 |  |
| 8061 | 8127 | tRNA-Lys |
| 8128 | 8128 |  |
| 8129 | 8289 | ATP8 |
| 8290 | 8329 | ATP8, ATP6 |
| 8330 | 8969 | ATP6 |
| 8970 | 8970 | ATP6, COX3 |
| 8971 | 9753 | COX3 |
| 9754 | 9773 | COX3, tRNA-Gly |
| 9774 | 9822 | tRNA-Gly |
| 9823 | 10169 | ND3 |
| 10170 | 10179 | ND3, tRNA-Arg |
| 10180 | 10238 | tRNA-Arg |
| 10239 | 10528 | ND4L |
| 10529 | 10535 | ND4L, ND4 |
| 10536 | 11906 | ND4 |
| 11907 | 11953 | ND4, tRNA-His |
| 11954 | 11976 | tRNA-His |
| 11977 | 12036 | tRNA-Ser |
| 12037 | 12037 |  |
| 12038 | 12108 | tRNA-Leu |
| 12109 | 13912 | ND5 |
| 13913 | 13929 | ND5, ND6 |
| 13930 | 14440 | ND6 |
| 14441 | 14509 | tRNA-Glu |
| 14510 | 14513 |  |
| 14514 | 15653 | CYTB |
| 15654 | 15726 |  |
| 15657 | 15725 | tRNA-Thr |
| 15726 | 15726 | tRNA-Thr, tRNA-Pro |
| 15727 | 15791 | tRNA-Pro |
| 15792 | 16338 | D-loop |

Base positions are according to the *Bos taurus* reference mtDNA genome sequence (GenBank accession no. V00654)
